# Neurological Involvement in Adult‐Onset Secondary Hemophagocytic Lymphohistiocytosis: Clinical Features and Prognostic Implications

**DOI:** 10.1002/brb3.71228

**Published:** 2026-01-29

**Authors:** Xue Wang, Yingying Zhao, Yanfei Han, Yongbo Zhang

**Affiliations:** ^1^ Department of Neurology Capital Medical University Affiliated Beijing Friendship Hospital Beijing China

**Keywords:** central nervous system, cytokine, neuroinflammatory, secondary hemophagocytic lymphohistiocytosis

## Abstract

**Background:**

Secondary hemophagocytic lymphohistiocytosis (sHLH) with central nervous system (CNS) involvement poses significant diagnostic and therapeutic challenges. This study aimed to characterize the clinical features, laboratory profiles, and prognostic impact of neurological involvement in adult‐onset sHLH.

**Methods:**

We analyzed 130 adult sHLH patients, comparing 28 with CNS involvement to 102 without neurological manifestations. Clinical parameters, neuroimaging, cerebrospinal fluid (CSF) profiles, cytokine levels, treatment responses, and survival outcomes were evaluated.

**Results:**

Patients with CNS involvement were older (median age, 54 vs. 46 years; *p* = 0.013) and had higher disease severity (median HScore, 250 vs. 210; *p* < 0.001). Malignancy‐associated sHLH was more prevalent in the CNS‐positive group (42.9% vs. 29.4%; *p* = 0.038). Neurological manifestations included altered mental status, impaired consciousness, and seizures. Neuroimaging revealed abnormalities in 71.4% of the cases, primarily T2‐weighted fluid‐attenuated inversion recovery hyperintensities and leptomeningeal enhancement. CNS‐positive patients exhibited markedly elevated inflammatory markers, most notably CSF Interleukin‐6 (*p* < 0.001). In multivariable analysis adjusted for malignancy, age, ferritin, and HScore, CNS involvement independently predicted mortality (adjusted HR = 2.0, 95% CI: 1.1–3.7, *p* = 0.023), with a significantly shorter median overall survival (6.5 vs. 11.5 months, *p* < 0.0001). Malignancy‐associated etiology and HScore ≥ 250 were also independent prognostic factors. The DEP (dexamethasone, etoposide, and polyethylene glycol‐asparaginase) regimen achieved a faster median time to initial response than the HLH‐94 protocol (9 vs. 14 days, *p* = 0.02).

**Conclusions:**

CNS involvement defines a severe phenotype of adult‐onset sHLH, characterized by malignancy‐prone etiology, intense neuroinflammation, and poor prognosis. We establish CNS involvement as an independent predictor of mortality, underscoring the critical need for early recognition and CNS‐directed therapies.

## Graphical abstract

This retrospective study showed that central nervous system (CNS) involvement in adult‐onset secondary hemophagocytic lymphohistiocytosis (sHLH) defines a severe phenotype and presented as an independent predictor of mortality. This study highlights the critical need for early neurological assessment and the development of CNS‐targeted treatment strategies.

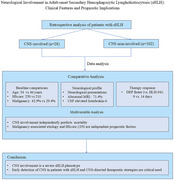

## 1 Introduction

Hemophagocytic lymphohistiocytosis (HLH) is a life‐threatening hyperinflammatory syndrome characterized by uncontrolled immune activation and multi‐organ dysfunction (Padhi et al. 2013; Janka 2024; Cox et al. 2024). HLH is categorized into primary and secondary forms based on etiology. Primary HLH results from specific genetic defects, whereas secondary HLH (sHLH) is triggered by factors such as infections, malignancies, or autoimmune diseases (R. Zhang, Cui, et al. 2022; Ramachandran et al. 2017; Stepp et al. 1999).

Although less frequent than pediatric‐onset cases, adult‐onset sHLH poses considerable clinical challenges due to its heterogeneous etiology and often acute presentation. Among its systemic complications, neurological involvement is increasingly recognized as a critical determinant of morbidity and mortality (Sivaramalingam et al. 2023; Radmanesh et al. 2020); however, it remains under‐recognized and insufficiently characterized. Neurological manifestations, including altered mental status, seizures, headache, and focal deficits, may mimic other central nervous system (CNS) disorders, complicating diagnosis (Radmanesh et al. 2020; Kam et al. 2023; Benallegue et al. 2021). The pathophysiology of neurological involvement in sHLH is not fully elucidated but is thought to involve direct viral effects, inflammatory cytokine release, and immune‐mediated injury (Horne et al. 2017). This clinical variability underscores the need for heightened diagnostic vigilance.

Previous studies have seldom provided comprehensive clinical characterization of adult patients with CNS involvement in sHLH (Song et al. 2018; Fohle et al. 2021), and data on associated diagnostic challenges and prognostic implications remain notably scarce. A clearer understanding of the clinical features of neurological involvement in adult‐onset sHLH is essential to improve diagnostic accuracy, guide therapy, and enhance outcomes.

This study aims to systematically analyze the clinical characteristics, diagnostic difficulties, and prognostic impact of neurological involvement in adult‐onset sHLH. By identifying key clinical patterns, we seek to provide insights that may aid clinicians in earlier recognition and more effective management of this serious complication.

## 2 Methods

### 2.1 Study Design and Patient Population

We conducted a retrospective cohort study of adult patients (≥ 18 years) diagnosed with sHLH with CNS involvement between January 2020 and December 2024 at Capital Medical University Affiliated Beijing Friendship Hospital. The diagnosis of sHLH required meeting at least five of eight HLH‐2004 diagnostic criteria (Henter et al. 2007). CNS involvement was defined by (1) presence of neurological symptoms (altered mental status, seizures, or focal deficits) and (2) either cerebrospinal fluid (CSF) abnormalities (pleocytosis > 5 cells/µL or protein > 45 mg/dL) or characteristic magnetic resonance imaging (MRI) findings (T2/fluid‐attenuated inversion recovery [FLAIR] hyperintensities or leptomeningeal enhancement). Informed consent was waived due to the retrospective nature of the study.

### 2.2 Data Collection and Outcomes

The data were extracted on demographics, clinical characteristics, laboratory parameters, treatment details, response assessments, neurological recovery, and survival outcomes. Demographic and clinical variables included age, sex, underlying etiology, and pre‐diagnostic interval (defined as the time from the onset of the first documented symptoms to the establishment of a definitive sHLH diagnosis). Serial laboratory measurements comprised ferritin, soluble CD25 (sCD25), CSF interleukin‐6 (IL‐6), and other inflammatory markers. Treatment regimens were categorized as either the DEP protocol (dexamethasone, etoposide, and polyethylene glycol‐asparaginase) or the HLH‐94 protocol (initial therapy with dexamethasone and etoposide, followed by guideline‐based maintenance therapy). Treatment response was classified as complete response, defined as the normalization of all HLH‐related biomarkers and the resolution of neurological symptoms, or partial response, defined as ≥ 50% improvement in at least three key parameters. Neurological recovery was evaluated using the modified Rankin Scale, the Mini‐Mental State Examination, and seizure frequency. Survival was measured from treatment initiation until death or the last documented follow‐up.

### 2.3 Statistical Analysis

Statistical analyses were performed using the SPSS software (version 22.0; IBM Corp., Armonk, NY, USA). Overall survival was defined as the time from treatment initiation to death from any cause or the last follow‐up. Continuous variables, which were non‐normally distributed, are presented as medians with interquartile ranges (IQRs). Categorical variables are expressed as frequencies and percentages. Group comparisons for the non‐normally distributed continuous data were made using the Mann–Whitney *U* test, while categorical variables were analyzed with Fisher's exact test or the chi‐square test, as appropriate. Survival curves were constructed using the Kaplan–Meier method, and differences between groups were assessed with the log‐rank test. Variables with a log‐rank *p* value <0.15 in univariate analysis were entered into a multivariate Cox proportional hazards regression model. Multicollinearity among covariates in the final Cox model was evaluated using variance inflation factors (VIFs); all VIFs were below 3.0, indicating no substantial multicollinearity. A two‐sided *p* value <0.05 was considered statistically significant.

## 3 Results

### 3.1 Baseline Characteristics of sHLH Patients With and Without CNS Involvement

The study cohort comprised 130 adult‐onset sHLH patients, including 28 (21.5%) with CNS involvement and 102 (78.5%) without neurological manifestations. Comparative analysis revealed significant differences between the groups (Table 1). Patients with CNS involvement were older (median age, 54 vs. 46 years; *p* = 0.013). The pre‐diagnostic interval was significantly longer in patients with CNS involvement (median 21 vs. 14 months, *p* = 0.002). This prolonged interval may reflect a more insidious onset of the underlying conditions (e.g., occult malignancies) in patients destined to develop neurological complications. Sex distribution was similar between the groups.

**TABLE 1:** Baseline characteristics of sHLH patients with and without CNS involvement.

Clinical characteristic	CNS (+) (*n* = 28)	CNS (−) (*n* = 102)	*p* value
Age (years), median (IQR)	54 (42–67)	46 (30–60)	0.013^a^
Male sex, *n* (%)	16 (57.1)	54 (52.9)	0.68^b^
Pre‐diagnostic interval (months)	21 (14–30)	14 (7–21)	0.002^a^
Underlying cause, *n* (%)			0.038^c^
Infection	8 (28.6)	50 (49.0)	
Malignancy	12 (42.9)	30 (29.4)	
Autoimmune diseases	4 (14.3)	16 (15.7)	
Unknown	4 (14.3)	6 (5.9)	
HScore, median (IQR)	250 (220–280)	210 (180–240)	< 0.001^a^

Abbreviations: CNS, central nervous system; sHLH, secondary hemophagocytic lymphohistiocytosis.

^a^ Mann–Whitney *U* test.

^b^ Chi‐square test.

^c^ Fisher's exact test.

Crucially, the etiological distribution differed significantly between the groups (*p* = 0.038). Malignancy was more prevalent as the underlying cause in patients with CNS involvement (42.9% vs. 29.4%), whereas infection‐triggered sHLH was more frequent in those without CNS manifestations (49.0% vs. 28.6%). The proportion of autoimmune diseases was similar. Notably, patients with neurological involvement presented with significantly higher HScore values (median 250 vs. 210, *p* < 0.001), reflecting greater overall disease severity at presentation.

### 3.2 Neurological Manifestations and Diagnostic Features in CNS‐Involved sHLH

Among 28 sHLH patients with CNS involvement, neurological symptoms were pervasive (Table 2). Altered mental status was the most frequent presentation (78.6%, *n* = 22), followed by impaired consciousness (64.3%, *n* = 18). Epileptic seizures occurred in 28.6% (*n* = 8), while meningeal signs (17.9%, *n* = 5) and focal deficits (21.4%, *n* = 6) were frequently observed.

**TABLE 2:** Clinical and paraclinical features of CNS involvement in sHLH patients.

Feature	sHLH CNS (+) (*n* = 28)
Neurological symptoms, *n* (%)	
Impaired consciousness	18 (64.3)
Epilepsy	8 (28.6)
Altered mental status	22 (78.6)
Meningeal signs	5 (17.9)
Focal neurological deficits	6 (21.4)
MRI abnormalities, *n* (%)	20/28 (71.4)
T2/FLAIR hyperintensity	15/20 (75.0)
Leptomeningeal enhancement	7/20 (35.0)
CSF findings, *n* (%)	
Protein elevation (> 45 mg/dL)	18/28 (64.2)
Pleocytosis (> 5 cells/µL)	12/28 (42.9)

Abbreviations: CNS, central nervous system; CSF, cerebrospinal fluid; MRI, magnetic resonance imaging; sHLH, secondary hemophagocytic lymphohistiocytosis.

MRI abnormalities were detected in 71.4% of the evaluated cases (*n* = 20/28), predominantly featuring T2/FLAIR hyperintensities (75.0%, *n* = 15/20). Leptomeningeal enhancement was observed in 35.0% (*n* = 7/20). CSF analysis revealed elevated protein levels (64.2%, *n* = 18/28; > 45 mg/dL) and modest pleocytosis (42.9%, *n* = 12/28; > 5 cells/µL).

### 3.3 Laboratory Profiles of sHLH Patients With and Without CNS Involvement

Patients with CNS involvement exhibited significantly more pronounced cytopenias and inflammatory activity compared to those without neurological manifestations (Table 3). Hemoglobin levels were lower in the CNS‐positive group (median 8.2 g/dL vs. 9.0 g/dL, *p* = 0.038), with more severe thrombocytopenia (median 35 × 10^9^/L vs. 60 × 10^9^/L, *p* = 0.003). A trend toward leukopenia was observed (median 2.8 × 10^9^/L vs. 3.5 × 10^9^/L, *p* = 0.051).

**TABLE 3:** Laboratory profiles of sHLH patients with and without CNS involvement.

Parameter, median (IQR)	CNS (+) (*n* = 28)	CNS (−) (*n* = 102)	*p* value
Routine blood tests			
Hemoglobin (g/dL)	8.2 (7.0–9.5)	9.0 (7.8–10.2)	0.038*
Platelets (×10^9^/L)	35 (20–60)	60 (40–85)	0.003*
WBC (×10^9^/L)	2.8 (1.5–4.0)	3.5 (2.0–5.2)	0.051
Inflammatory markers			
Ferritin (µg/L)	18,200 (12,000–35,000)	10,800 (7500–20,000)	< 0.001*
sCD25 (U/mL)	6800 (4500–9200)	4700 (3000–6500)	0.001*
CRP (mg/L)	85 (50–120)	60 (30–90)	0.009*
Cytokines (serum)			
IL‐6 (pg/mL)	140 (90–200)	60 (30–100)	< 0.001*
IFN‐γ (pg/mL)	350 (200–500)	200 (100–350)	0.002*
CSF‐specific markers			
CSF protein (mg/dL)	120 (80–200)	40 (30–60)	< 0.001*
CSF IL‐6 (pg/mL)	250 (150–400)	30 (20–50)	< 0.001*
Pleocytosis (cells/µL)	15 (8–32)	2 (1–4)	< 0.001*

Abbreviations: CNS, central nervous system; CSF, cerebrospinal fluid; MRI, magnetic resonance imaging; sHLH, secondary hemophagocytic lymphohistiocytosis; WBC, white blood cell.

*p < 0.05, Mann–Whitney *U* test.

Markers of systemic hyperinflammation were significantly elevated in patients with CNS involvement: serum ferritin (median, 18,200 vs. 10,800 µg/L; *p* < 0.001), sCD25 (median, 6,800 vs. 4700 U/mL; *p* = 0.001), and C‐reactive protein (CRP; median, 85 vs. 60 mg/L; *p* = 0.009). Serum cytokine analysis revealed substantially higher levels of IL‐6 (median, 140 vs. 60 pg/mL; *p* < 0.001) and interferon‐γ (IFN‐γ; median, 350 vs. 200 pg/mL; *p* = 0.002) in the CNS‐positive group.

CSF analysis demonstrated robust discriminative features. CNS‐involved cases had elevated protein levels (median 120 mg/dL vs. 40 mg/dL, *p* < 0.001), pleocytosis (median 15 cells/µL vs. 2 cells/µL, *p* < 0.001), and markedly higher CSF IL‐6 concentrations (median 250 pg/mL vs. 30 pg/mL, *p* < 0.001).

### 3.4 Impact of CNS Involvement on Survival

**FIGURE 1:**
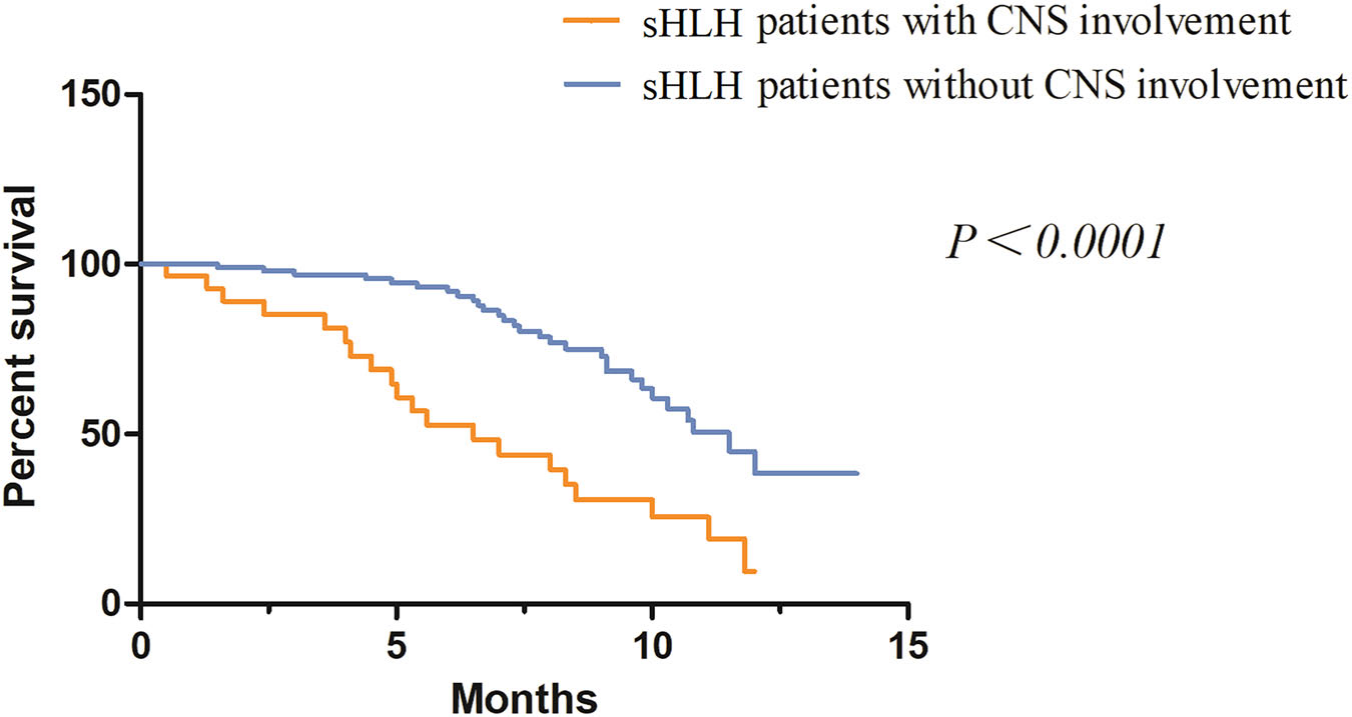
Kaplan–Meier survival analysis between patients with and without CNS involvement.

Survival outcomes were significantly worse in patients with CNS involvement. The median overall survival was 6.5 months in the CNS‐positive group compared to 11.5 months in the CNS‐negative group (p < 0.0001, log‐rank test; Figure 1). The 1‐year survival rate was markedly lower in patients with CNS involvement (28.6% vs. 62.3%).

Multivariable Cox proportional hazards analysis showed that in the unadjusted model, CNS involvement was associated with a substantially increased mortality risk (hazard ratio [HR] = 3.1, 95% confidence interval [CI]: 1.8–5.3). After adjustment for age, ferritin level, HScore, and underlying etiology (classified as malignancy‐associated vs. other), CNS involvement remained a significant and independent predictor of mortality (adjusted HR = 2.0, 95% CI: 1.1–3.7, *p* = 0.023; Table 4). No significant multicollinearity was detected among the covariates in the final model (all variance inflation factors <3.0), supporting the robustness of the regression estimates. Malignancy‐associated etiology was also independently associated with poor survival (adjusted HR = 2.4, 95% CI: 1.3–4.4, *p* = 0.005). An HScore ≥ 250 was another independent prognostic factor (adjusted HR = 2.2, 95% CI: 1.2–4.2, *p* = 0.014).

**TABLE 4:** Multivariate cox regression analysis of survival predictors.

Variable	Unadjusted HR (95% CI)	Adjusted HR (95% CI)^a^	*p* value
CNS involvement	3.1 (1.8–5.3)	2.0 (1.1–3.7)	0.023
Age > 50 years	1.9 (1.1–3.2)	1.4 (0.8–2.5)	0.28
Ferritin > 15,000 µg/L	2.3 (1.3–4.0)	1.8 (1.0–3.3)	0.052
HScore ≥ 250	2.7 (1.5–4.9)	2.2 (1.2–4.2)	0.014
Malignancy‐associated etiology	2.8 (1.6–4.8)	2.4 (1.3–4.4)	0.005

Abbreviations: CI, confidence interval; CNS, central nervous system; HR, hazard ratio. ^a^Adjusted model: Adjusted for all variables listed in the table (CNS involvement, Age > 50 years, Ferritin > 15,000 µg/L, HScore ≥ 250, and Malignancy‐associated etiology).

### 3.5 Treatment Efficacy and Neurological Outcomes in CNS‐Involved sHLH Patients

Among the 28 sHLH patients with CNS involvement, 39.3% (*n* = 11) achieved a complete response and 25.0% (*n* = 7) attained a partial response (Table 5). The median time to response was significantly shorter with the DEP regimen than with the HLH‐94 protocol (9 vs. 14 days; *p* = 0.02).

**TABLE 5:** Treatment efficacy and neurological outcomes in sHLH patients with CNS involvement.

Parameter	Overall (*n* = 28)	DEP protocol (*n* = 15)	HLH‐94 protocol (*n* = 13)	*p* value
Treatment response				
Complete response (CR), *n* (%)	11 (39.3)	7 (46.7)	4 (30.8)	0.46^a^
Partial response (PR), *n* (%)	7 (25.0)	4 (26.7)	3 (23.1)	1.00^a^
Time to response (days)^c^, median (IQR)	11 (8–16)	9 (7–12)	14 (10–18)	0.02^b^
Neurological recovery, *n* (%)				
mRS improvement (Δ ≥ 2)^d^	11 (39.3)	8 (53.3)	3 (23.1)	0.13^a^
Seizure freedom	6/8 (75.0)	4/5 (80.0)	2/3 (66.7)	0.99^a^
Cognitive recovery^e^	8/22 (36.4)	4 /8 (50)	3/8 (37.5)	0.23^a^
Biomarker dynamics, *n* (%)				
CSF IL‐6 decline > 50%^f^	16 (57.1)	10 (66.7)	6 (46.2)	0.27^a^
Ferritin normalization^g^	13 (46.4)	9 (60.0)	4 (30.8)	0.15^a^
Survival outcomes, *n* (%)				
6‐Month survival	16 (57.1)	11 (73.3)	5 (38.5)	0.07^a^
1‐Year survival	8 (28.6)	6 (40.0)	2 (15.4)	0.23^a^

^a^ Fisher's exact test.

^b^ Mann–Whitney *U* test.

^c^Time from treatment initiation to first observed response (CR/PR). ^d^Modified Rankin scale improvement from baseline to 3‐month follow‐up. ^e^MMSE score increase ≥ 3 points or clinician‐assessed improvement. ^f^CSF IL‐6 reduction > 50% from baseline at Day 14. ^g^Ferritin < 500 µg/L by Day 28.

Neurological improvement, defined as a reduction in the modified Rankin Scale score of ≥ 2 points, was observed in 39.3% (*n* = 11) of the patients, with no significant difference between the treatment groups. Seizure freedom was achieved in 75.0% (6/8) of the affected patients, and cognitive recovery occurred in 36.4% (8/22); neither outcome differed significantly between the DEP and HLH‐94 groups. Regarding biomarker dynamics, the proportions of patients achieving a > 50% reduction in CSF IL‐6 or normalization of ferritin did not differ significantly between the treatment regimens (*p* = 0.27 and *p* = 0.15, respectively).

The 6‐month survival rate was 57.1% (*n* = 16), with a notable but nonsignificant advantage for DEP treatment (73.3% vs. 38.5%, *p* = 0.07). One‐year survival did not differ significantly between the 2 treatment groups.

## 4 Discussion

This study provides a comprehensive analysis of the clinical features, diagnostic challenges, and prognostic implications of neurological involvement in adult‐onset sHLH. Our findings underscore the significance of CNS involvement in sHLH outcomes.

Consistent with previous reports (Georgiadou et al. 2019; Harada et al. 2021), patients with CNS involvement in our cohort were older and exhibited a longer interval from documented symptoms to the diagnosis of sHLH. Patients with CNS involvement in our cohort were older and experienced a longer diagnostic delay. The distinct etiological profile observed, characterized by a higher prevalence of malignancy‐associated sHLH in CNS‐positive patients, aligns with prior studies linking malignancy to severe neurological manifestations in sHLH (Löfstedt et al. 2024; Johnson et al. 2024; Zoref‐Lorenz et al. 2022). This prolonged diagnostic interval likely reflects the insidious onset of underlying conditions, particularly occult malignancies, which may present with non‐specific symptoms before culminating in fulminant sHLH and neurological complications. This association necessitates careful clinical evaluation for CNS involvement, particularly in older patients and those with underlying malignancies.

Neurological symptoms, predominantly altered mental status and impaired consciousness, were highly frequent in our cohort with CNS involvement, reflecting similar findings reported by H.‐Q. Zhang, Yang, et al. (2022). Neuroimaging results, including frequent T2/ FLAIR hyperintensities and leptomeningeal enhancement, support the notion that these findings reflect underlying inflammatory processes within the CNS (Lehrer et al. 2021; Borda et al. 2024; Dean et al. 2023). The variability in CSF findings, such as elevated protein levels and pleocytosis, further highlights the importance of comprehensive CSF analysis to diagnose CNS involvement and differentiate sHLH from other neurological conditions, such as autoimmune encephalitis or CNS infections (Shyu et al. 2021; Horne et al. 2008; Kim et al. 2012).

Patients with CNS involvement exhibited significantly elevated levels of systemic inflammatory markers, including ferritin and sCD25. This observation aligns with the hypothesis that heightened inflammatory responses correlate with greater disease severity (Kim et al. 2012; Weaver and Behrens 2014; Varshney et al. 2024). The strong association between elevated IL‐6 levels and CNS involvement reinforces its potential role as a biomarker of disease activity and a target for therapeutic intervention in sHLH (Wang et al. 2024).

A core finding of this study is that CNS involvement independently predicts mortality in adult‐onset sHLH. Even after adjusting for key confounders—including age, disease severity (HScore), hyperferritinemia, and the presence of underlying malignancy, CNS involvement remained significantly associated with increased mortality risk. This result is further supported by sensitivity analyses within etiology‐defined subgroups, confirming that the adverse prognosis associated with CNS disease extends beyond its association with malignancy‐prone etiologies. It underscores that the neurological syndrome itself, likely driven by severe neuroinflammation, is a major contributor to poor outcomes (Kim et al. 2012; Zhao et al. 2018). These findings emphasize the need to incorporate neurological status into initial risk stratification.

Regarding therapeutic management, our data suggest that the DEP regimen may offer a faster time to initial response. Although rates of biomarker response and neurological improvement were numerically higher in the DEP group, these differences did not reach statistical significance. Nevertheless, given the established efficacy of DEP in other challenging HLH contexts, such as Epstein–Barr virus–associated HLH (Lai et al. 2018), and the clinical imperative for rapid cytoreduction in CNS‐involved disease, this regimen warrants further investigation. The observed rates of seizure freedom and neurological improvement confirm that therapeutic intervention can mitigate certain neurological sequelae, although the variable cognitive recovery highlights an area requiring dedicated study and potential rehabilitation strategies.

Several limitations should be considered.The retrospective, single‐center design may introduce bias, and the sample size, particularly of the CNS‐positive subgroup, limits statistical power for detailed treatment comparisons and generalizability. Future prospective, multicenter studies employing standardized neurological assessments are needed to validate our findings, elucidate the underlying neuroimmunological mechanisms, and optimize CNS‐directed therapeutic approaches.

## 5 Conclusion

In summary, CNS involvement defines a severe and distinct phenotype in adult‐onset sHLH, characterized by a malignancy‐prone etiology, intense systemic and neuroinflammatory responses, and a poor prognosis. We establish CNS involvement as an independent predictor of mortality, with elevated CSF IL‐6 underscoring a key neuroinflammatory mechanism. While early aggressive therapy (e.g., the DEP regimen) may accelerate initial response, overall survival remains guarded. These findings mandate the integration of neurological assessment into early diagnostic workflows and underscore the urgent need for novel, CNS‐directed therapeutic strategies in sHLH.

## Author Contributions

X.W. wrote the main manuscript text. Y.F.H. collected the data. Y.Y.Z. analyzed the data and prepared the figures. Y.B.Z. designed the experiment and revised the main manuscript text. All authors reviewed the manuscript.

## Funding

This study was supported by National Natural Science Foundation of China (82501733).

## Conflicts of Interest

The authors declare no conflicts of interest.

## Data Availability

The datasets analyzed in the present study are available from the corresponding author on reasonable request.
